# Supplementary material for: The interactome of intact mitochondria by cross-linking mass spectrometry provides evidence for coexisting respiratory supercomplexes
Source: Mol Cell Proteomics. 2017 Dec 8;17(2):216–32. doi: 10.1074/mcp.RA117.000470 (PMC5795388; doi:10.1074/mcp.RA117.000470)
Supplement: Supplemental Data [file supp_RA117.000470_133922_0_supp_23978_fzffwf.zip › spectra_annotation/mito_DR_spectra_annotation/424-1-7-1-8-1.pdf]

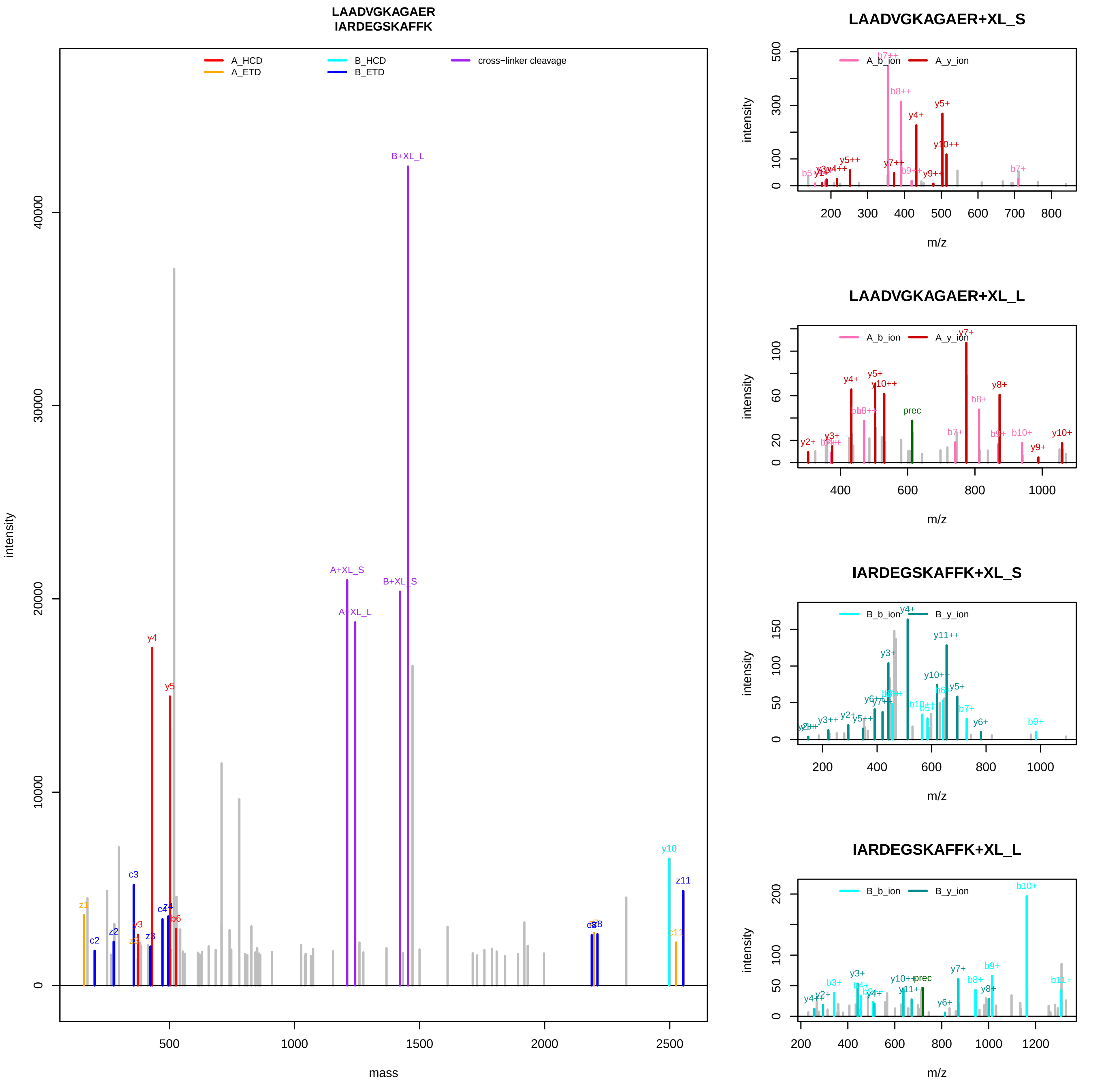

LAADVGKAGAER
IARDEGSKAFFK
A_HCD
A_ETD
B_HCD
B_ETD
cross−linker cleavage
B+XL_L
A+XL_S
B+XL_S
A+XL_L
y4
y5
c3
y10
z11
c11
c2
z2
z1
intensity
mass
0
10000
20000
30000
40000
500
1000
1500
2000
2500
LAADVGKAGAER+XL_S
A_b_ion
A_y_ion
b7++
b8++
y5+
y4+
y10++
y5++
y7++
b7+
LAADVGKAGAER+XL_L
y7+
y4+
y5+
y10++
y8+
b8+
prec
b10+
y10+
y9+
y2+
y3+
IARDEGSKAFFK+XL_S
B_b_ion
B_y_ion
y4+
y11++
y3+
y10++
y5+
b7+
y6+
b9+
IARDEGSKAFFK+XL_L
b10+
b9+
y7+
b11+
b3+
y3+
y10++
prec
y8+
b8+
y6+
m/z
intensity
